# Effects of Grape Seed Proanthocyanidins with Different Polymerization Degrees on the Phenolic Compounds and Sensory Quality of Cabernet Sauvignon Wine During Bottle Aging

**DOI:** 10.3390/foods15091512

**Published:** 2026-04-27

**Authors:** Yilan Zhang, Qiuyu Zhang, Junyi Liu, Yunxuan Nan, Xiaoyu Cheng, Yulin Fang, Xiangyu Sun, Junjun Li

**Affiliations:** 1 College of Enology, Northwest A&F University, Yangling 712100, China; zylatte@163.com (Y.Z.); 13335453855@163.com (Q.Z.); liujunyi_5325@163.com (J.L.); yunxuan8421@163.com (Y.N.); freesia0603@163.com (X.C.); fangyulin@nwsuaf.edu.cn (Y.F.); 2 Shaanxi Provincial Key Laboratory of Viti-Viniculture, Yangling 712100, China

**Keywords:** grape seed proanthocyanidins, degree of polymerization, wine, phenolic compounds, color characteristics, astringency

## Abstract

Wine phenolic composition is strongly influenced by tannin structure, yet how the polymerization degree of exogenous proanthocyanidins modulates wine quality during aging remains unclear. This study investigated the effects of adding grape seed proanthocyanidins (GSP) with different mean degrees of polymerization (mDP 4.63, 3.29, and 1.31) to Cabernet Sauvignon wine by analyzing phenolic compounds, tannin structure, anthocyanin components, CIELAB color parameters, and astringency over 6 months of bottle aging. Low-mDP GSP (rich in galloylated monomers) provided the biggest initial phenolic boost, while high-mDP GSP (dominated by non-galloylated units) sustained tannin enrichment throughout aging. Low-mDP GSP accelerated tannin maturation and color evolution toward aged wine characteristics, with Mv-3-Coglu identified as a key precursor for brick-red hue development. Sensory evaluation revealed that high-mDP GSP enhanced coarse and drying astringency, whereas low-mDP GSP promoted velvety mouthfeel. These findings establish that GSP polymerization degree critically determines phenolic evolution, color stability, and mouthfeel during bottle aging, providing a scientific basis for selecting structure-specific proanthocyanidins to achieve targeted wine quality outcomes.

## 1. Introduction

Proanthocyanidins, also known as condensed tannins, are among the most abundant phenolic compounds in grapes and play a decisive role in determining the sensory quality of wine [1]. Structurally, these polymers are composed of flavan-3-ol units, with grape seed proanthocyanidins (GSPs) predominantly belonging to the B-type and existing as mixtures of oligomers (degree of polymerization, DP 2-4) and polymers (DP > 4) [2]. During winemaking, proanthocyanidins are extracted from grape seeds and transferred into wine, where they substantially influence astringency, bitterness, color stability, and aging potential [3].

The perception of proanthocyanidins in terms of mouthfeel is essentially mediated through their interactions with the oral environment. Astringency is not considered a basic taste but rather a tactile sensation involving dryness, roughness, tightening, and puckering of the oral mucosa. The currently accepted mechanism posits that proanthocyanidins bind to and precipitate proline-rich proteins (PRPs) in saliva, thereby disrupting the lubricating salivary film that normally protects the oral epithelium. This loss of lubrication generates the characteristic drying and rough mouthfeel associated with astringency [4]. Importantly, the efficiency of this interaction depends on the structural features of the proanthocyanidins. Compared to smaller oligomers, larger polymeric proanthocyanidins exhibit stronger binding affinity for PRPs and induce more extensive protein precipitation [5]. Thus, in the context of red wine, astringency intensity is primarily driven by polyphenol concentration, degree of polymerization, and degree of galloylation. Beyond astringency, proanthocyanidins also play a critical role in modulating wine color stability. In young red wines, copigmentation contributes over 30% of the visible color, a process in which anthocyanins are stabilized through non-covalent interactions with colorless copigments, including flavanols and proanthocyanidins [6]. During bottle aging, anthocyanins undergo direct condensation reactions with proanthocyanidins, forming covalent polymeric pigments. These polymeric pigments are resistant to sulfur dioxide bleaching and oxidative degradation, thereby contributing significantly to the color of aged red wines [7]. The degree of polymerization of proanthocyanidins critically influences these color stabilization mechanisms. Malien-Aubert et al. (2002) demonstrated [8], using model wine solutions, that increasing the degree of polymerization improves the color stability of anthocyanins, with the catechin tetramer exhibiting a marked stabilizing effect. In contrast, the monomer promoted the formation of flavylium salts, leading to a yellowish solution. Therefore, understanding how proanthocyanidins with different polymerization degrees influence wine attributes is of fundamental enological importance.

Given their significant role in wine quality, the exogenous addition of tannins has been explored as a strategy for wine enhancement. However, most existing studies have focused on commercial tannin preparations, which are typically ill-defined mixtures with broad and heterogeneous DP distributions [9]. While these studies have reported various effects on phenolic compounds and sensory properties, the complexity of commercial products makes it difficult to attribute observed outcomes to specific structural features [10]. Moreover, research on tannin addition has often been limited to isolated quality parameters rather than systematically assessing the holistic impact on wine phenolic profiles and sensory characteristics. To date, there remains a lack of systematic investigation into how GSPs with precisely controlled polymerization degrees affect the overall phenolic composition and sensory quality of red wines.

Therefore, the present study aimed to investigate the effects of GSPs with varying mean degrees of polymerization (mDP) on the phenolic composition, chromatic characteristics, and sensory attributes of Cabernet Sauvignon wine during bottle aging. GSP fractions with distinct mDP were prepared from grape pomace using a green degradation approach and introduced into finished wine, thereby isolating polymerization degree as the key variable. By integrating phenolic compound analysis, colorimetric evaluation, and astringency assessment, this study seeks to establish a comprehensive understanding of how proanthocyanidin structure modulates wine quality. The findings are expected to fill a critical theoretical gap regarding the structure-activity relationship of proanthocyanidins in wine matrices and to provide a scientific foundation for precision-oriented quality improvement, while also contributing to the valorization of winemaking byproducts.

## 2. Materials and Methods

### 2.1. Materials

Grape pomace was obtained from Xinjiang Bainianzhuang Winery Co., Ltd. (Xinjiang, China). The base wine used in this study was the dry red wine Cabernet Sauvignon (vintage 2024) provided by Inner Mongolia Yangguang Tianyu Wine Co., Ltd. (Inner Mongolia, China), which was produced without the addition of exogenous tannins.

### 2.2. Chemicals and Reagents

Methanol and acetonitrile (LC-MS grade) were purchased from Xilong Scientific Co., Ltd. (Shantou, China). Ethanol and hydrochloric acid (analytical reagent grade) were obtained from Sichuan Xilong Scientific Co., Ltd. (Chengdu, China). Acetone (analytical grade) was purchased from Chengdu Kelong Chemical Co., Ltd. (Chengdu, China). Catechin (HPLC grade) and vanillin (analytical grade) were sourced from Shanghai Yuanye Biotechnology Co., Ltd. (Shanghai, China). Epicatechin, epicatechin gallate, and epigallocatechin (all HPLC grade) were purchased from Shanghai Aladdin Biochemical Technology Co., Ltd. (Shanghai, China). Phloroglucinol (analytical grade) was obtained from Fuchen (Tianjin) Chemical Reagent Co., Ltd. (Tianjin, China). Ascorbic acid (analytical grade) was purchased from Xilong Scientific Co., Ltd. (Shantou, China).

### 2.3. Preparation of GSP with Different Polymerization Degrees

Grape seed proanthocyanidins (GSP) were extracted and purified based on the method of Chen et al. [11] with minor modifications. Briefly, grape pomace was thoroughly washed and dried in an oven at 40 °C. Grape seeds were manually separated from skins and collected, then ground and sieved through 40–60 mesh screens (0.250–0.425 mm). The resulting powder was defatted with n-hexane to remove lipophilic impurities, then extracted with 70% (*v*/*v*) ethanol in the dark for 12 h. Following centrifugation, the supernatant was concentrated under reduced pressure and freeze-dried to yield crude GSP. Crude extracts were further purified using an AB-8 macroporous resin column (16–60 mesh) eluted with 60% (*v*/*v*) aqueous acetone. To obtain fractions with varying mDP, the purified GSP was dissolved in deionized water, homogenized using a high-speed disperser, and transferred to a high-pressure reactor. The reactor headspace was purged with nitrogen to ensure an inert atmosphere. Reactions were conducted at predetermined temperatures and durations. Upon cooling, the solutions were concentrated and freeze-dried to obtain GSP samples with varying average degrees of polymerization. The samples were stored at −20 °C until analysis.

### 2.4. Determination of Phenolic Compounds in Prepared GSP

Phenolic compounds in the prepared GSP samples were quantified by ultra-performance liquid chromatography coupled with triple quadrupole tandem mass spectrometry (UPLC-QQQ-MS/MS) following a previously described method [12] with minor modifications. Analyses were performed on an Exion LC AD UPLC system (SCIEX, Framingham, MA, USA) coupled with a Triple Quad 5500+ mass spectrometer (SCIEX, Framingham, MA, USA) using a Kinetex C18 column (100 mm × 2.1 mm, 1.7 μm) maintained at 40 °C. The mobile phase consisted of 0.1% formic acid in water (A) and acetonitrile (B) at a flow rate of 0.4 mL/min, with an injection volume of 2 μL. The gradient elution program was as follows: 0–3 min, 5% B; 3–6 min, 20% B; 6–12 min, 45% B; 12–18 min, 100% B; 18–20 min, 5% B. Mass spectrometric detection was performed in negative ion mode with the following parameters: curtain gas, 30 psi; ion spray voltage, 4.5 kV; ion source temperature, 550 °C; Gas 1, 60 psi; Gas 2, 50 psi. All analyses were performed in triplicate.

### 2.5. Determination of the Mean Degree of Polymerization (mDP) of Grape Seed Proanthocyanidins

The mDP of GSP fractions was determined by phloroglucinolysis according to Kennedy et al. [13] with minor modifications. GSP samples were dissolved in methanol (10 mg/mL). An aliquot (100 μL) was mixed with 100 μL of phloroglucinol reagent (0.1 N HCl in methanol containing 50 g/L phloroglucinol and 10 g/L ascorbic acid) and incubated at 50 °C for 20 min. The reaction was terminated with 1.0 mL of 40 mM sodium acetate buffer. After filtration (0.22 μm), the samples were analyzed by UPLC at 280 nm. The mDP was calculated as the molar ratio of total flavan-3-ol units to terminal units. All analyses were performed in triplicate.

### 2.6. Preparation of Red Wines with the Addition of GSPs

GSP samples with different mean degrees of polymerization (mDP) were prepared as described in Section 2.3. Based on their mDP values determined in Section 2.5, three GSP samples representing high, medium, and low polymerization degrees were selected for wine addition experiments. The addition level (0.6 g/L) was determined according to Kovac et al. [14]. The selected samples and their corresponding preparation conditions are presented in Table 1.

After the GSP addition, all wine samples were bottled and stored at 15–18 °C in the dark for 6 months. Samples were collected at 0, 1, 3, and 6 months of aging for subsequent analysis of phenolic compounds, tannin structure and composition, anthocyanin composition, color parameters, and astringency evaluation. Wine samples were labeled as follows: samples supplemented with high-polymerization degree grape seed proanthocyanidins (GSP-P) were designated as Group P; those supplemented with medium-polymerization degree proanthocyanidins (GSP-120A) were designated as Group PM; and those supplemented with low-polymerization degree proanthocyanidins (GSP-150B) were designated as Group PL. For wine samples of a specific aging month, taking P-1M as an example, it represents the wine sample with GSP-P added that has been aged for 1 month, and so on. At each sampling time point, three independent bottles from each treatment group were randomly selected and analyzed in triplicate.

### 2.7. Determination of Phenolic Compounds in Wine

#### 2.7.1. Total Tannin Content

Total tannin (TT) content was determined by the hydrochloric acid-vanillin method according to Cáceres-Mella et al. [15] with minor modifications. A 4% (*w*/*v*) vanillin-methanol solution was prepared. An aliquot (0.5 mL) of the wine sample was mixed with 3 mL of vanillin-methanol solution and 1.5 mL of concentrated HCl, then incubated in the dark at room temperature for 20 min. Absorbance was measured at 510 nm against a blank (methanol replacing vanillin-methanol solution) using a spectrophotometer (Shanghai MAPADA Instruments Co., Ltd. Shanghai, China). Results were expressed as mg catechin equivalents per liter (mg CE/L). All analyses were performed in triplicate.

#### 2.7.2. Total Phenolic Content

Total phenolic (TP) content was determined by the Folin–Ciocalteu method according to Lazarova et al. [16] with minor modifications. Wine samples were diluted 10-fold with deionized water. An aliquot (0.2 mL) of the diluted sample was sequentially mixed with 5 mL of deionized water, 0.5 mL of Folin–Ciocalteu reagent, and 1.5 mL of 20% Na_2_CO_3_. The mixture was brought to a final volume of 10 mL with deionized water and incubated in the dark for 1 h. Absorbance was measured at 765 nm. Results were expressed as gallic acid equivalents (mg GAE/L). All analyses were performed in triplicate.

#### 2.7.3. Total Flavonoid Content

Total flavonoid (TFO) content was determined according to Kalinowska et al. [17] with minor modifications. An aliquot (0.1 mL) of wine was sequentially mixed with 0.9 mL of methanol and 2.7 mL of 30% methanol. After thorough mixing, 0.2 mL of NaNO_2_ solution (0.5 mol/L) and 0.2 mL of AlCl_3_ solution (0.3 mol/L) were added. The mixture was allowed to stand for 5 min, then 1 mL of NaOH solution (1 mol/L) was added and mixed thoroughly. Absorbance was measured at 510 nm against a blank (AlCl_3_ replaced with deionized water). Results were expressed as rutin equivalents (mg RE/L). All analyses were performed in triplicate.

#### 2.7.4. Total Flavan-3-ol Content

Total flavan-3-ol (TF) content was determined according to Meng et al. [18] with minor modifications. An aliquot (0.1 mL) of wine was mixed with 3 mL of HCl-methanol solution (1 mol/L) containing 0.1% p-DMACA and allowed to react at room temperature for 10 min. Absorbance was measured at 640 nm against methanol as a blank. Results were expressed as (+)-catechin equivalents (mg CE/L). All analyses were performed in triplicate.

#### 2.7.5. Total Anthocyanin Content

Total anthocyanin (TA) content was determined by the pH differential method according to Lee et al. [19] with minor modifications. KCl buffer (pH 1.0) was prepared by dissolving 1.86 g of KCl in 980 mL of deionized water and adjusting the pH to 1.0 with concentrated HCl. Sodium acetate buffer (pH 4.5) was prepared by dissolving 54.43 g of CH_3_COONa·3H_2_O in 960 mL of deionized water and adjusting the pH to 4.5 with concentrated HCl.

An aliquot of the wine sample was diluted with each buffer and allowed to equilibrate in the dark for 15–20 min. Absorbance was measured at 520 nm and 700 nm within 23–40 min using a spectrophotometer. All analyses were performed in triplicate. Total anthocyanin content was calculated as follows:

A = (A520 − A700)_pH=1.0_ − (A520 − A700)_pH=4.5_ W = (A × MW × DF × 1000)/ε

where MW is the molecular weight of malvidin-3-glucoside (493.5 g/mol), DF is the dilution factor (20), and ε is the molar extinction coefficient of malvidin-3-glucoside (28,000).

### 2.8. Analysis of Tannin Structure and Composition in Wine

#### 2.8.1. Tannin Structure Analysis

The structural analysis of condensed tannins in wine samples was performed according to Tu et al. [20] with minor modifications. Sample preparation, phloroglucinolysis, and UPLC conditions were the same as those described in Section 2.5, except that wine samples were subjected to SPE pretreatment prior to analysis. Briefly, a C18 SPE cartridge (1 g, 6 mL) was preconditioned with 10 mL of methanol followed by 10 mL of distilled water. An aliquot (5 mL) of the wine sample was loaded by gravity. The cartridge was washed with 25 mL of distilled water, and tannins were eluted with 10 mL of methanol. The eluate was evaporated to dryness under reduced pressure at 35 °C, and the residue was redissolved in 1.5 mL of methanol for phloroglucinolysis. All analyses were performed in triplicate.

#### 2.8.2. Tannin Composition Analysis

Individual tannin components in wine were analyzed according to Tu et al. [21] with minor modifications. Wine samples were filtered through a 0.22 μm membrane filter and directly analyzed by HPLC. The instrumentation, column, and detection wavelength were the same as those described in Section 2.8.1. The mobile phase consisted of 1% formic acid in water (A) and 1% formic acid in acetonitrile (B) at a flow rate of 0.8 mL/min. The injection volume was 20 μL, and the column temperature was maintained at 35 °C. The gradient elution program was as follows: 0–10 min, 8% B; 10–20 min, 15% B; 20–40 min, 20% B; 40–45 min, 80% B; 45–50 min, 80% B; 50–56 min, 8% B. All analyses were performed in triplicate.

### 2.9. Analysis of Anthocyanin Composition in Wine

Monomeric anthocyanins in wine were analyzed by HPLC according to Wei et al. [22] with minor modifications. Wine samples were filtered through a 0.22 μm membrane filter prior to analysis. Separation was performed on an LC-20A HPLC system (Shimadzu, Kyoto, Japan) equipped with a photodiode array detector, using the same column as described in Section 2.8. The mobile phase consisted of formic acid/acetonitrile/water (1:4:32, *v*/*v*/*v*) as solvent A and formic acid/acetonitrile/water (1:20:16, *v*/*v*/*v*) as solvent B at a flow rate of 1.0 mL/min. The injection volume was 20 μL, and the column temperature was maintained at 35 °C. The gradient elution program was as follows: 0–45 min, 35% B; 45–46 min, 100% B; 46–50 min, 100% B; 50–51 min, 0% B; 51–55 min, 0% B. Detection was at 520 nm. Results were expressed as malvidin-3-O-glucoside equivalents. All analyses were performed in triplicate.

### 2.10. Color Analysis of Wine

Color analysis was performed using a wine color analyzer (Haineng, Jinan, China) based on the CIELab system, as described by Ricci et al. [23], with minor modifications. In this system, L* represents lightness (0 = black, 100 = white), a* represents the red-green balance (positive values indicate red, negative values indicate green), and b* represents the yellow-blue balance (positive values indicate yellow, negative values indicate blue). Hue angle (h), chroma (C*), and total color difference (ΔE*ab) were calculated as follows:

$$\begin{array}{c} \mathrm{Hue}=\arctan(b^{*}/a^{*})\\ \mathrm{Chroma}=\sqrt{a^{*2}+b^{*2}}\\ ∆E^{*}ab=\sqrt{{\Delta L}^{*2}+{\Delta a}^{*2}+{\Delta b}^{*2}} \end{array}$$

### 2.11. Astringency Evaluation

This study was conducted in accordance with the Declaration of Helsinki. Informed consent was obtained from all subjects involved in the study. All participants were provided with a detailed ingredient list and confirmed that they had no allergies to the components prior to the sensory evaluation.

Sensory evaluation was conducted by a trained panel of 13 assessors (8 females, 5 males; aged 20–26 years) from the College of Enology, Northwest A&F University. Astringency perception was rated on a 5-point categorical scale (1 = very weak, 2 = weak, 3 = medium, 4 = relatively strong, 5 = strong), and the astringency descriptive terms used are shown in Table 2.

Prior to evaluation, panelists underwent training sessions to calibrate their perception of wine mouthfeel. After the wine samples had aged for the specified time, they were taken from the cellar for astringency evaluation. A total of four tasting sessions were conducted in this experiment: immediately after GSP addition (0 months of aging), and after 1, 3, and 6 months of aging. Wine samples were prepared in duplicate, each coded with a three-digit random number, and presented in random order within each set. Astringency evaluation was performed using ISO 3591 standard tasting glasses. Blind evaluations were conducted in two sessions, with each sample evaluated for no more than 5 min. Sessions were conducted in a professional tasting room equipped with individual booths, spittoons, and rinsing stations. Between samples, panelists rinsed with purified water and cleansed their palates with unsalted soda crackers. Results were expressed as M values calculated as follows:

$$M=\sqrt{F\times I}$$

where *F* is the frequency of citation for each sensory descriptor, and *I* is the ratio of the mean intensity to the maximum possible intensity [24,25].

### 2.12. Data Analysis

All experiments were performed in triplicate, and results were expressed as mean ± standard deviation. Data were analyzed using SPSS Statistics 26.0 (IBM, Chicago, IL, USA). Statistical significance was determined by one-way analysis of variance (ANOVA) followed by Tukey’s test (*p* < 0.05). Sensory astringency data were analyzed using a Linear Mixed Model (LMM) with assessor as a random factor and wine sample and repetition as fixed factors (*p* < 0.05) to verify panel consistency and sample differences. Graphs, including bar charts, line graphs, cluster heatmaps, correlation heatmaps, and radar charts, were generated using Origin 2025 (OriginLab Corporation, Northampton, MA, USA).

## 3. Results and Discussion

### 3.1. Characterization of GSP with Different Polymerization Degrees

GSP fractions with varying mean degrees of polymerization (mDP) were obtained by high-pressure reactor treatment under different temperature-time combinations. The mDP values of the fractions were determined by UPLC after phloroglucinolysis, and the recovery rates are presented in Table 3.

Prior to treatment, the mDP of purified GSP (GSP-P) was 4.63 ± 0.43. After high-pressure treatment, mDP decreased progressively with increasing temperature and time, ranging from 3.92 ± 0.08 to 1.31 ± 0.02. The recovery rates ranged from 86.98% to 92.40%, indicating that the high-pressure reactor treatment effectively produced GSP fractions with distinct polymerization degrees while maintaining acceptable yields. Based on these results, three fractions with significantly different mDP values were selected for subsequent wine addition experiments: GSP-P (mDP = 4.63 ± 0.43) representing high polymerization degree (when added to the wine sample, it is denoted as P), GSP-120A (mDP = 3.29 ± 0.06) representing medium polymerization degree (when added to the wine sample, it is denoted as PM), and GSP-150B (mDP = 1.31 ± 0.02) representing low polymerization degree (when added to the wine sample, it is denoted as PL).

### 3.2. Phenolic Profiles of GSP with Different Polymerization Degrees

**Figure 1:**
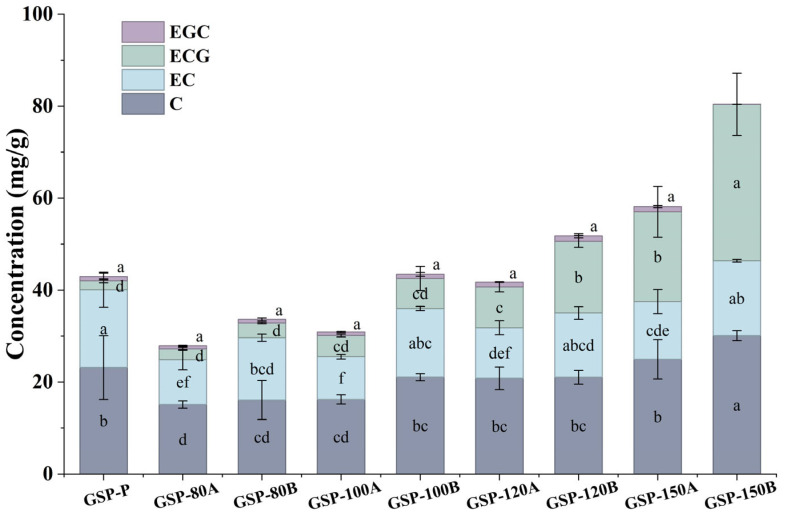
Content of monomer procyanidins in GSP samples of different treatment groups. C: Catechin; EC: Epicatechin; ECG: Epicatechin-gallate; EGC: Epigallocatechin. Different lowercase letters on the same color block indicate significant differences in the substance among different samples (*p* < 0.05); EGC was not detected in the GSP-150B group.

The phenolic composition of GSP fractions was analyzed by UPLC-QQQ-MS/MS, with results presented in Table 4 and Figure 1. High-pressure reactor treatment significantly altered the phenolic profiles of GSP in a temperature- and time-dependent manner. The contents of monomeric flavan-3-ols varied markedly among fractions. The GSP-150B fraction, which had the lowest mDP (1.31), exhibited the highest contents of catechin (C, 30.11 mg/g) and epicatechin gallate (ECG, 34.03 mg/g), while GSP-P (mDP 4.63) showed the highest epicatechin (EC) content (16.94 mg/g). Notably, ECG increased progressively as mDP decreased, suggesting preferential cleavage of galloylated terminal units under severe treatment conditions [26]. This increase in ECG contributed substantially to the depolymerization of polymeric proanthocyanidins. In contrast, epigallocatechin (EGC) was detected at low levels (<1.2 mg/g) in all fractions, consistent with its predominant occurrence in grape skins rather than seeds [27].

Regarding oligomeric proanthocyanidins, procyanidin B3 was the most abundant dimer, with the highest content (9.96 mg/g) observed in GSP-80B (Table 4). The contents of B1 and B3 were more sensitive to treatment conditions than B2 and B4, showing greater variation among fractions. Trimer content decreased significantly under severe conditions, falling below 0.5 mg/g in 150 –treated samples, thereby contributing to the observed decrease in mDP. Procyanidin A2, an A-type proanthocyanidin, was detected at low levels (<1 mg/g) in all fractions, confirming the predominance of B-type proanthocyanidins in grape seeds [28]. These results demonstrate that high-pressure reactor treatment not only reduces the polymerization degree of GSP but also systematically alters its phenolic composition, particularly by increasing galloylated monomers and decreasing oligomeric proanthocyanidins.

### 3.3. Evolution of Tannin Polymerization Degree (mDP) During Bottle Aging

**Figure 2:**
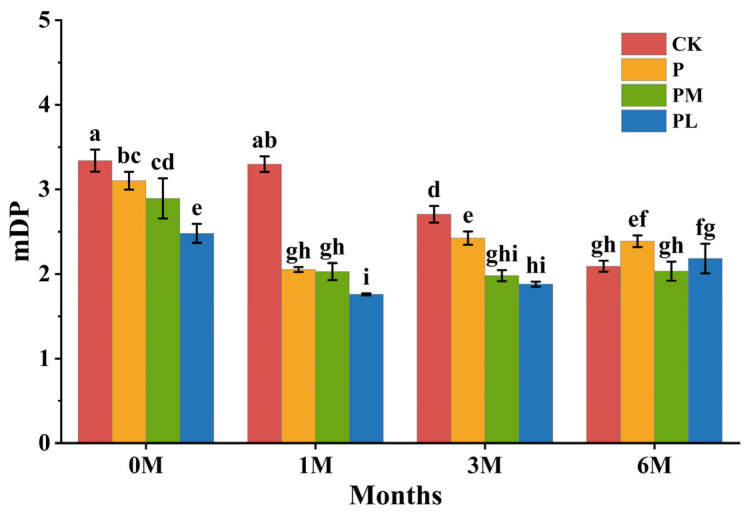
mDP of wine samples at different aging periods. CK refers to the blank control group without GSP addition; P refers to the group with GSP-P addition; PM refers to the group with GSP-120A addition; PL refers to the group with GSP-150B addition. Different lowercase letters indicate significant differences (*p* < 0.05).

Across the different aging periods, mDP of tannins in the wine samples ranged from 1.7 to 3.5 (Figure 2). Overall, the mDP exhibited a trend of initial decrease followed by stabilization or a slight increase, consistent with the range reported in previous studies [29]. In the control group, the mDP was the highest during the first three months of aging and gradually decreased over time, declining from approximately 3.35 to 2.09. Except for the control group, the mDP of the wine samples with GSP added showed a significant decrease after the initial aging period of 1 month. During subsequent aging, the mDP of the P group samples recovered and stabilized, the mDP of the PM group samples remained essentially stable, and the mDP of the PL group samples slightly increased. From the above, it can be seen that the addition of GSP can significantly reduce the initial mDP of the wine samples and cause it to decrease at a faster rate. It can achieve the aging effect that the control group wine samples would only achieve after a much longer aging period in a shorter time.

### 3.4. Analysis of Total Phenolic Compounds in Wines Supplemented with GSP During Bottle Aging

**Figure 3:**
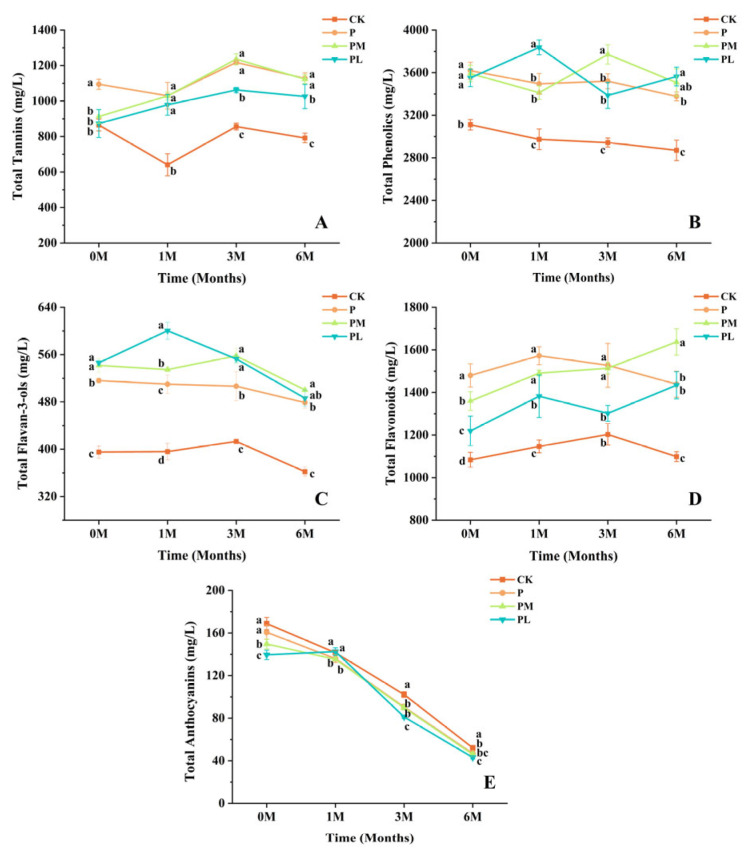
Changes in phenolic composition of wines during bottle aging. (**A**) Total tannins, (**B**) Total phenolics, (**C**) Total flavan-3-ols, (**D**) Total flavonoids, and (**E**) Total anthocyanins in control and GSP-supplemented wines over 6 months of aging. Data are expressed as mean ± SD (*n* = 3). In each line chart, different lowercase letters in the same column (same month) indicate significant differences (*p* < 0.05).

The evolution of phenolic compounds in wines supplemented with GSP fractions of different polymerization degrees was monitored over 6 months of bottle aging, and the results are presented in Figure 3. Throughout the aging period, all GSP-supplemented wines maintained higher levels of total phenolics, total tannins, total flavan-3-ols, and total flavonoids than the control, whereas total anthocyanins showed the opposite trend. At 0 months, the control group exhibited 3110.37 ± 48.72 mg/L total phenolics, whereas P, PM, and PL groups showed 3618.33 ± 78.70, 3591.67 ± 77.66, and 3550.83 ± 81.42 mg/L, respectively. The PL group reached the highest total phenolics (3837.78 ± 69.35 mg/L) and total flavan-3-ols (600.4 ± 14.5 mg/L) at 1 month, coinciding with its lowest mDP (1.3), which facilitated direct release of flavan-3-ol monomers. Typically, low-molecular-weight proanthocyanidins tend to exhibit higher solubility and hydrophilicity. GSP-150B shows greater solubility in hydroethanolic matrices, making it more readily released and quantifiable during the early stages of wine aging. In contrast, highly polymerized fractions are more likely to form larger aggregates or interact more strongly with macromolecular components in wine, thereby reducing their measurable concentration at the initial stage of aging. The PM group exhibited sustained advantages at later stages, showing the highest total tannins (1235.8 ± 30.4 mg/L) at 3 months and the highest total flavonoids (1637.1 ± 61.7 mg/L) at 6 months, indicating that medium-polymerization GSP provides prolonged phenolic enhancement. The underlying mechanism of this phenomenon is that the degree of polymerization (DP) of GSP exerts a non-linear regulation on its binding stability and detectability within the wine matrix (proteins, polysaccharides) [30]. Low-oligomeric GSP (mDP ≈ 1.3) is readily released initially but tends to dissociate at later stages. Highly polymeric GSP (mDP ≈ 4.6) can form stable complexes, but its net contribution decreases due to reduced solubility and steric hindrance [31]. In contrast, medium-polymerization GSP (mDP ≈ 3.3) lies exactly within the “optimal window”: it forms stable complexes with the matrix to delay degradation while remaining effectively detectable by the total tannin and total flavonoid assay methods.

Total anthocyanin contents were consistently lower in GSP-supplemented wines than in the control throughout aging. At 0 months, the control contained 168.82 ± 5.69 mg/L, while P, PM, and PL groups showed progressively lower values of 160.67 ± 6.80, 149.70 ± 4.54, and 139.48 ± 4.40 mg/L, respectively. This may be attributed to the fact that the added GSP provides new solid surfaces that physically adsorb free anthocyanins in the wine. In addition, the flavanol molecules in GSP may form intermolecular complexes with anthocyanins. Both effects together reduce the apparent extraction yield of free anthocyanins. Despite these initial differences, all groups exhibited similar rates of decline during aging, with reductions exceeding 70% by 6 months. The control decreased to 52.12 ± 2.28 mg/L, while P, PM, and PL groups decreased to 46.76 ± 2.68, 46.01 ± 1.68, and 43.08 ± 1.35 mg/L, respectively. This is because, although GSP acts as a copigment, its effect is weak, and copigmentation itself does not significantly slow down the natural oxidative degradation rate of anthocyanins [32]. More importantly, the addition of GSP does not disrupt the chemical system of the wine to accelerate degradation. Instead, it participates in the formation of more stable polymeric pigments (e.g., flavanol-anthocyanin adducts), thereby increasing the total amount of pigments [33]. These results demonstrate that the polymerization degree of added GSP differentially modulates phenolic evolution during wine aging: low-mDP GSP (GSP-150B) provides the highest initial phenolic boost, medium-mDP GSP (GSP-120A) offers sustained enhancement. Meanwhile, all GSP additions reduce initial anthocyanin extractability without accelerating their subsequent degradation.

### 3.5. Evolution of Flavan-3-ol Monomers During Bottle Aging

The changes in six flavan-3-ol monomers (GC, EGC, C, EC, EGCG, and ECG) during bottle aging were monitored, and the results are presented in Table 5. The addition of GSP with different polymerization degrees significantly influenced both the initial release and subsequent evolution of these monomers in wine, revealing distinct structure-dependent patterns.

Immediately after GSP addition, all supplemented wines exhibited higher levels of C and EC compared to the control, confirming that GSP directly contributes these monomers to the wine matrix. Notably, the distribution of monomers varied markedly with mDP: the P group (mDP 4.6) showed the highest C (133.55 mg/L) and EC (77.65 mg/L) contents, while the PL group (mDP 1.3) displayed the highest EGCG (19.34 mg/L) and ECG (5.08 mg/L). This differential pattern suggests that high-mDP GSP, with its more extensive polymer network, preferentially releases terminal non-galloylated units (C and EC) upon initial incorporation into wine. In contrast, low-mDP GSP, having undergone extensive depolymerization during high-pressure treatment, contains abundant galloylated monomers (EGCG and ECG) that are readily released into the wine matrix [34]. The detectability of ECG only in the PM and PL groups at 0 months further supports this interpretation, as galloylated units are known to be more prevalent in oligomeric proanthocyanidins [35].

As aging progressed, C and EC contents declined in all groups, but GSP-supplemented wines consistently maintained higher levels than the control. Among them, the P group exhibited a relatively slower decline, with C and EC decreasing by approximately 13.2% and 15.9% within the first three months, respectively, compared to declines of 28.1% and 28.3% in the control group, demonstrating greater stability. This enhanced stability may be attributed to the formation of colloidal structures by high-mDP GSP, which can physically entrap and protect monomers from oxidative degradation [36]. The gradual emergence of ECG in the P group during aging, from undetectable levels at 0 months to 8.88 mg/L at 6 months, provides compelling evidence for this mechanism. This indicates that high-mDP GSP undergoes slow, sustained hydrolysis in the acidic wine environment, releasing ECG as a cryptic reservoir that becomes available only over time [37]. In contrast, the PL group maintained consistently high EGCG levels (17.23–21.00 mg/L) throughout aging with minimal fluctuation, reflecting the inherent resistance of galloylated monomers to oxidation once released. The PM group exhibited intermediate behavior, with moderate initial monomer levels and notable stability in EGC at later stages (133.28 mg/L at 6 months).

These results demonstrate that the polymerization degree of GSP profoundly affects not only the quantity but also the temporal dynamics of flavan-3-ol monomers in wine. High-mDP GSP functions as a slow-release system, providing sustained C, EC, and particularly ECG over extended aging, which may be advantageous for long-term wine aging where gradual tannin evolution is desired. Low-mDP GSP offers immediate enrichment of galloylated monomers, potentially enhancing early mouthfeel attributes such as velvety astringency. Medium-mDP GSP provides a balanced contribution, combining immediate availability with sustained release. These structure-dependent release patterns provide a mechanistic basis for the selective use of GSP fractions to achieve targeted wine quality outcomes during bottle aging.

### 3.6. Changes in Tannin Structure and Subunit Composition During Bottle Aging

The structural characteristics of tannins in wine samples during bottle aging were analyzed by phloroglucinolysis-HPLC, and the results are presented in Table 6. GSP addition preserves total tannin content during aging. Throughout the 6-month aging period, all GSP-supplemented wines except the PL-0M group maintained higher total condensed tannin levels than the control. At 0 months, the P group (mDP 4.6) showed the highest value (422.72 mg/L), followed by PM (332.94 mg/L) and PL (263.00 mg/L), compared to the control (303.73 mg/L). By 6 months, the P and PM groups remained elevated (386.07 and 372.98 mg/L, respectively), while the PL group declined to 286.47 mg/L, approaching control levels (251.86 mg/L). These results indicate that higher mDP GSP provides more sustained enrichment of wine tannins, whereas low-mDP GSP undergoes faster integration and loss through precipitation or degradation [38].

GSP supplementation alters subunit composition and enhances galloylation. Tannins in all samples were predominantly composed of procyanidins (%PC 61.58–85.47%), with prodelphinidins (%PD 7.81–19.54%) constituting a minor proportion. During aging, %PC decreased in all groups, reflecting progressive depolymerization, but GSP-supplemented wines showed slower decline rates and maintained higher %PC than the control. The degree of galloylation (%G) exhibited the most striking differences. At 0 months, %G in the PM (7.74%) and PL (8.81%) groups was substantially higher than in the control (1.47%) and P (1.33%) groups, consistent with their elevated ECG levels (Table 5). As aging progressed, %G increased across all groups, reaching 12.80% in the control, 8.61% in P, 13.84% in PM, and 9.52% in PL at 6 months. This universal increase reflects the selective retention of galloylated subunits due to their higher chemical stability during tannin degradation. Notably, the PL-0M group exhibited mDP (2.48) and %G (8.81%) values already approaching those of CK-6M (2.09 and 12.80%), demonstrating that low-mDP GSP can accelerate the structural evolution typically achieved only after extended aging.

Terminal and extension subunits reveal where structural changes occur. Analysis of subunit composition showed distinct behaviors between terminal and extension positions. In terminal subunits, catechin (tC) dominated initially (24.65–35.07%) but was rapidly replaced by epicatechin (tEC), which remained predominant thereafter (17.74–26.70% at 6M). Epicatechin gallate (tECG) increased significantly during aging, particularly in GSP-supplemented groups, reaching 10.65–17.29% at 6 months, supporting the notion that galloylated subunits are more resistant to further depolymerization once exposed at terminal positions [35]. Epigallocatechin (tEGC) remained consistently low or undetectable, confirming that this subunit rarely occupies terminal positions in grape seed-derived tannins [39]. In extension subunits, epicatechin (exEC) was consistently dominant (25.79–82.77%), reflecting the fundamental structure of grape seed proanthocyanidins. Exogenous GSP had minimal impact on extension subunit distribution, with exC and exECG showing low proportions and no clear trends. This indicates that GSP addition primarily influences terminal subunit profiles and galloylation patterns, while extension subunits remain largely determined by the original tannin structure.

These results demonstrate that GSP supplementation modulates wine tannin evolution through three distinct mechanisms. High-mDP GSP provides sustained enrichment of total tannin content, maintaining elevated levels throughout aging. All GSP additions enhance galloylation through selective retention of stable subunits, with medium-mDP GSP showing the most pronounced effect. Low-mDP GSP accelerates structural maturation, rapidly achieving mDP and %G profiles characteristic of aged wines. The limited impact on extension subunits suggests that tannin evolution during aging is primarily driven by cleavage events at terminal positions rather than by wholesale restructuring of the polymer backbone. These findings provide a mechanistic basis for the strategic selection of GSP fractions with different polymerization degrees to achieve targeted tannin profiles during bottle aging.

### 3.7. Changes in Monomeric Anthocyanin Composition During Bottle Aging

The content and composition of monomeric anthocyanins in wine samples during bottle aging were analyzed by HPLC, and the results are presented in Table 7. Nine monomeric anthocyanins were quantified, including five non-acylated monoglucosides (Dp-3-Glu, Cy-3-Glu, Pt-3-Glu, Pn-3-Glu, Mv-3-Glu) and four acylated derivatives (Pn-3-Acglu, Mv-3-Acglu, Pn-3-Coglu, Mv-3-Coglu). The addition of GSP with different polymerization degrees significantly influenced the initial extractability and the subsequent evolution of these anthocyanins during aging.

At 0 months, the control group exhibited the highest contents of all individual anthocyanins, with malvidin-3-O-glucoside (Mv-3-Glu) reaching 102.49 mg/L, accounting for 62.81% of the total monomeric anthocyanins. Compared to the control, Mv-3-Glu content was 13.67% lower in the P group, 15.65% lower in the PM group, and 20.86% lower in the PL group, with similar trends observed for other anthocyanins. This initial reduction can be attributed to the formation of co-pigmentation complexes between the added proanthocyanidins and anthocyanins through hydrogen bonding or hydrophobic interactions, which either reduce extraction efficiency or alter the spectral characteristics of monomeric anthocyanins. These findings are consistent with previous reports that seed-derived tannins reduce the extractable concentration of monomeric anthocyanins upon initial interaction [40].

During the 6-month aging period, the contents of all monomeric anthocyanins declined steadily across all groups, with reductions exceeding 80%. Mv-3-Glu, which had the highest initial content, decreased from 102.49 mg/L to 18.83 mg/L in the control group (81.62% reduction). In the GSP-supplemented groups, similar reduction rates were observed: 83.87% in P, 83.33% in PM, and 82.84% in PL. These results demonstrate that although GSP addition reduces the initial extractability of monomeric anthocyanins, it does not significantly accelerate or catalyze their subsequent degradation during wine aging. The added proanthocyanidins appear to affect only the initial interaction without promoting further anthocyanin loss.

The stability of individual anthocyanins varied significantly during aging. Cyanidin-3-O-glucoside (Cy-3-Glu), a non-acylated anthocyanin, steadily decreased and became undetectable in all groups by 6 months. In contrast, malvidin-3-O-(6-O-trans-p-coumaroyl)-glucoside (Mv-3-Coglu), an acylated derivative, showed an increasing trend during aging, reaching approximately 5 mg/L in all groups at 6 months, significantly higher than its initial values. This divergent behavior confirms that acylated anthocyanin monomers possess greater chemical stability than non-acylated forms, consistent with the stability order reported by Zhang et al. [41], where pyranoanthocyanins are the most stable, followed by acylated anthocyanins, while non-acylated monomers are the least stable. The p-coumaroylated structure of Mv-3-Coglu not only enhances its intrinsic stability but may also participate in ester exchange or polymerization reactions with other anthocyanins during later aging stages, leading to its relative accumulation.

These results demonstrate that GSP addition primarily affects the initial extractability of monomeric anthocyanins through co-pigmentation interactions, resulting in lower detected concentrations at the beginning of aging. However, the subsequent degradation rates remain unchanged, indicating that GSP does not promote anthocyanin loss during aging. In the context of dry red wine production, however, the quality benchmark is not simply the highest possible concentration of free monomeric anthocyanins, but rather the equilibrium between tannins and anthocyanins that governs both color stability and mouthfeel evolution during aging. The initial decrease detected in our study can be partially attributed to the formation of copigmentation complexes via non-covalent interactions (hydrogen bonding and hydrophobic stacking) between the added GSP and native anthocyanins. These complexes, while rendering the anthocyanins less extractable or spectroscopically detectable as free monomers, are known to protect the chromophore from nucleophilic attack by water or SO_2_, thereby enhancing short-to-medium term color stability [42]. More importantly, our finding that GSP addition did not accelerate the subsequent degradation of monomeric anthocyanins over the 6-month aging period strongly suggests that the added proanthocyanidins do not catalyze anthocyanin loss. Instead, they may participate in the gradual formation of more stable polymeric pigments (tannin-anthocyanin condensation products) and pyranoanthocyanins, which are the true determinants of long-term color evolution in aged red wines [43]. Furthermore, the higher stability of acylated anthocyanins (e.g., Mv-3-Coglu) observed in all groups, including GSP-supplemented ones, indicates that the intrinsic chemical structure remains the dominant factor for stability during aging. Therefore, while GSP supplementation transiently lowers the measured pool of free monomeric anthocyanins, it does not compromise—and may potentially benefit—the long-term color stability and structural palate of red wines by promoting a more stable pigment profile and contributing to tannin polymerization. This trade-off between initial monomeric anthocyanin extractability and eventual wine aging potential should be carefully considered depending on the winemaking goal: for wines intended for short-term consumption, preserving high initial monomeric anthocyanins may be preferred; for wines designed for aging, the addition of GSP could be a viable strategy to modulate tannin structure and enhance color stability without accelerating pigment degradation.

### 3.8. Changes in CIELab Color Parameters During Bottle Aging

**Figure 4:**
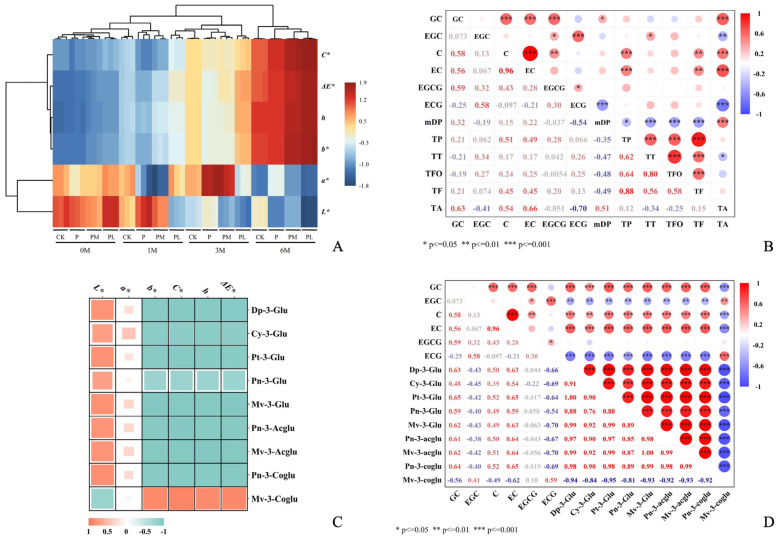
Comprehensive analysis of phenolic composition and color parameters in wine samples during aging. (**A**) Heatmap of CIELab parameters at different aging periods; (**B**) Correlation between flavan-3-ol monomers and mean degree of polymerization (mDP) with phenolic substances; (**C**) Heatmap of the correlation between CIELab parameters and monomeric anthocyanins; (**D**) Correlation between flavan-3-ol monomer composition and monomeric anthocyanins in the wine samples.

The CIELAB parameters provide a quantitative representation of wine color. Figure 4A presents a heatmap of the CIELAB parameters for the wine samples at different aging periods. The results show that the lightness (L*) of the wines fluctuated over time, with no consistent trends, while the red–green index (a*) increased slightly or remained stable from 0 to 3 months of aging, but decreased in all groups by 6 months. The yellow–blue index (b*) exhibited a consistent upward trend throughout aging, with values increasing most rapidly in the PL group, reflecting the typical “yellowing” that occurs in wine during aging. The continuous increase in chroma (C*) indicates an enhancement in color intensity across all groups. Meanwhile, the increase in the hue angle (h) represents the characteristic shift in wine color from purple–red toward brick red during the aging process. Total color difference (ΔE*) increased progressively in all groups, with the highest values observed in PL, followed by PM, P, and CK, indicating that lower mDP GSP accelerates overall color evolution. These results demonstrate that GSP addition modulates wine color development in a polymerization-dependent manner, with lower polymerization degrees promoting more rapid progression toward the color characteristics of aged wines.

### 3.9. Correlation Analysis

#### 3.9.1. Correlation Between Flavan-3-ol Monomers, mDP, and Phenolic Compounds

To investigate the relationships among flavan-3-ol monomers, mean degree of polymerization (mDP), and phenolic compounds, a correlation analysis was performed, with results presented in Figure 4B. Significant positive correlations were observed among most flavan-3-ol monomers, with the strongest correlation between C and EC, indicating highly consistent variation patterns. Both C and EC also showed strong positive correlations with total phenolics, total flavan-3-ols, and total anthocyanins, suggesting that catechin and epicatechin are core contributors to these phenolic fractions. In contrast, ECG and EGC exhibited negative correlations with total anthocyanins. The mDP showed a strong negative correlation with ECG, indicating that ECG predominantly exists in low-polymerized tannins or is readily released from highly polymerized tannins. Notably, The mDP is strongly negatively correlated with total tannin, total flavonoid, and total flavan-3-ol content, indicating that the longer the polymer chain (i.e., the higher the mDP), the lower the extractable or retainable amount. The positive correlation between mDP and total anthocyanins likely reflects tannin-anthocyanin complex formation that protects anthocyanins from degradation. These results demonstrate that catechin and epicatechin serve as key positive contributors to phenolic fractions, while galloylated monomers exhibit distinct negative associations with anthocyanins.

#### 3.9.2. Correlation Between CIELAB Parameters and Monomeric Anthocyanins

To investigate the contribution of individual anthocyanins to wine color, a correlation analysis was conducted between CIELAB parameters and monomeric anthocyanin contents, with results shown in Figure 4C. Most monomeric anthocyanins showed significant positive correlations with L* (lightness) and negative correlations with b* (yellowness), h (hue angle), and C* (chroma), indicating that higher anthocyanin concentrations enhance brightness while preserving a younger purple-red hue with lower yellow tones and saturation. In contrast, malvidin-3-O-(6-O-trans-p-coumaroyl)-glucoside (Mv-3-Coglu) exhibited opposite correlation patterns, with negative correlation with L* and positive correlations with b*, h, C*, and ΔE*. This demonstrates its unique role in promoting the characteristic shift toward brick-red hues during aging. Correlations with a* (redness) were generally low across all anthocyanins, consistent with the understanding that monomeric anthocyanins contribute indirectly to red color through conversion to polymeric pigments rather than directly [44]. These results confirm that while most anthocyanins maintain youthful color characteristics, Mv-3-Coglu serves as a key precursor for the color evolution typical of aged wines.

#### 3.9.3. Correlation Between Flavan-3-ol Monomers and Monomeric Anthocyanins

To investigate the relationship between flavan-3-ol monomers and monomeric anthocyanins, a Pearson correlation analysis was performed, with results shown in Figure 4D. GC, C, and EC exhibited moderate to strong positive correlations with most anthocyanins, except Mv-3-Coglu. These non-galloylated flavan-3-ols possess a free hydroxyl group at the C3 position, facilitating direct condensation with anthocyanins to form polymeric pigments, consistent with their role as key precursors for color evolution [43]. In contrast, EGC and ECG showed negative correlations with anthocyanins, while EGCG exhibited very weak correlations. Their additional substituents (trihydroxylated B-ring or galloyl group) increase steric hindrance, reducing interaction efficiency with anthocyanins. Galloylated monomers, such as ECG, tend to form metastable colloidal particles that are prone to aggregation and precipitation, which explains their reciprocal relationship with free anthocyanins [45]. Notably, Mv-3-Coglu again exhibited correlation patterns opposite to other anthocyanins, further confirming its unique interaction mode with flavanols. These results demonstrate that the structural features of flavan-3-ols critically determine their interactions with anthocyanins: non-galloylated forms actively participate in pigment formation, whereas galloylated forms exhibit competitive or aggregative behavior. This structure-dependent interaction pattern provides a mechanistic basis for understanding how GSP fractions with different polymerization degrees differentially modulate wine color evolution during bottle aging through their distinct profiles of released flavan-3-ol monomers.

### 3.10. Effect of GSP Addition on Wine Astringency

To examine differences among wine samples across various astringency sub-attributes and to ensure the reliability of the assessors, an LMM analysis was performed on the astringency sensory data. Taking “the velvety sensation of the first assessment “as an example, the fixed-effects test results showed that the main effect of wine sample was highly significant (F(3, 96) = 19.568, *p* < 0.001), indicating significant differences in velvety sensation among the wine samples; the main effect of repetition was not significant (F(1, 96) = 0.754, *p* = 0.387), suggesting no systematic bias between the two replicate assessments; and the interaction between wine sample and repetition was not significant (F(3, 96) = 0.534, *p* = 0.660), indicating that the pattern of changes across the two assessments was consistent for all wine samples. Similar results were obtained for the remaining astringency sub-attributes. These findings demonstrate that the sensory data collected in this study have high internal consistency and repeatability, and that the differences observed among wine samples based on these data are genuine and reliable.

**Figure 5:**
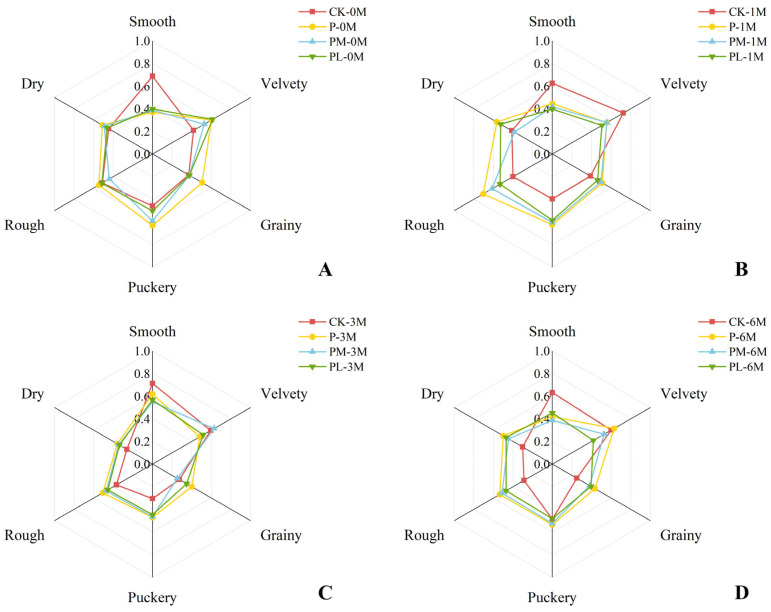
Sensory astringency radar plots of the wine samples. Panels (**A**–**D**) correspond to sensory evaluations at 0, 1, 3, and 6 months of aging, respectively.

The astringency characteristics of wine samples from each group at different aging periods (0, 1, 3, and 6 months) were evaluated, with results presented as radar plots in Figure 5. Immediately after GSP addition, all supplemented wines exhibited distinct astringency profiles compared to the control. The P group (high-mDP GSP, 4.6) showed the highest overall astringency, with top scores for grainy, puckery, rough, and drying attributes, while the difference between the PM and PL groups was not obvious. Group PL, however, stands out with the highest score in velvety texture. During aging, astringency intensity initially increased at 1 month, decreased by 3 months, indicating a shift from “rough” to “supple” perception, and showed a slight rebound at 6 months, possibly related to mDP recovery. Throughout aging, the control group consistently exhibited the smoothest mouthfeel. These results demonstrate that GSP polymerization degree critically determines astringency quality: highly polymerized GSP enhances coarse and drying sensations through stronger protein precipitation and larger aggregate formation [46]. Meanwhile, low-polymerized GSP promotes velvety mouthfeel via smaller, more uniform protein aggregates that form a lubricating layer on oral surfaces [47]. Soares S. et al. (2017) also found that as the degree of polymerization increases, the affinity between proanthocyanidins and proteins increases, and the size of the aggregates they form also increases, thereby generating a stronger sensation of friction and roughness in the oral cavity [48]. Quijada-Morín et al. (2014) further confirmed that oligomeric proanthocyanidins, due to their smaller molecular size and flexible conformation, tend to form a thin and uniform lubricating layer with salivary proteins in the oral cavity, thereby producing a velvety mouthfeel [49].

Interestingly, although the high-polymerization group (Group P) exhibited a more pronounced rough texture at the early stage of aging (0 and 1 month), as aging progressed, the roughness-related attribute scores of Group P gradually became comparable to those of Groups PM and PL. Moreover, after 6 months of aging, its velvety attribute score surpassed that of all other groups, while still maintaining a certain level in other astringency attributes. This may be related to the fact that tannin molecules further polymerize with one another under the action of trace oxygen to form larger complexes, reducing the amount of free tannins and coating or neutralizing the originally rough “sharp edges” [50]. The formation of stable complex pigments through the binding of tannins with anthocyanins is also one of the reasons; this process not only softens the astringency of tannins but also stabilizes the color of the wine [51]. In addition, tannins can bind with polysaccharides, proteins, and other substances in the wine, further weakening their direct interaction with salivary proteins, thereby reducing the rough astringency sensation and making the mouthfeel smoother [52]. This structure-dependent astringency characteristic provides a basis for enhancing wine body and directionally modulating wine mouthfeel by selecting appropriate GSP: for wines intended to improve soft astringency in the short term, adding GSP with a lower degree of polymerization may be preferable; for wines with aging potential, adding GSP with a higher degree of polymerization can not only strengthen the wine’s structure but also yield a smoother mouthfeel while maintaining the structure after aging.

## 4. Conclusions

This study demonstrates that the polymerization degree (mDP) of grape seed proanthocyanidins (GSP) acts as a dominant structural factor that regulates the evolution of phenolic composition, color stability, and sensory properties of Cabernet Sauvignon wine during bottle aging. GSP fractions with contrasting mDP values exerted structure-dependent effects on phenolic profiles, with low-mDP GSP rich in galloylated flavan-3-ols and high-mDP GSP dominated by non-galloylated units. During 6 months of bottle aging, all GSP treatments effectively preserved higher contents of total phenolics, tannins, flavan-3-ols, and flavonoids compared with the control wine. Specifically, low-mDP GSP induced a more pronounced initial increase in phenolic contents, whereas medium-mDP GSP provided sustained phenolic enhancement throughout aging. Despite a slight initial decrease in anthocyanin extractability due to co-pigmentation, GSP supplementation did not accelerate anthocyanin degradation, thereby improving color stability during bottle storage. Structural evolution analysis revealed that high-mDP GSP maintained stable tannin abundance, while low-mDP GSP accelerated the maturation of tannin structure, rapidly establishing the mDP and galloylation patterns typical of aged wines. Similarly, low-mDP GSP promoted a more typical aged wine color evolution, with malvidin-3-O-coumaroyl-glucoside (Mv-3-Coglu) confirmed as a key pigment contributor to the development of brick-red hues. Therefore, for wines intended for aging, the addition of GSP is more suitable, as it can enhance color stability without promoting pigment degradation. Sensory evaluation further confirms that during short-term aging, the mean degree of polymerization (mDP) exerts a more direct effect on astringency modulation: GSP with high mDP enhances roughness and dryness through stronger protein precipitation, whereas GSP with low mDP, by forming smaller and more uniform protein aggregates, delivers a superior velvety mouthfeel. As for wines designed for aging, the addition of high-polymerization GSP can enhance wine body while simultaneously making the astringency smoother. Collectively, these results establish a clear structure–function relationship between GSP polymerization degree and wine aging evolution. By selecting GSP fractions with tailored mDP values, the phenolic composition, color development, and mouthfeel of red wine can be directionally modulated during bottle aging. These findings provide a scientific basis and practical strategy for the targeted improvement of aging quality in red wine using structure-specific proanthocyanidins, and future studies may extend this approach to longer aging periods and diverse grape varieties to further validate its industrial applicability.

## Figures and Tables

**Table 1:** Samples and Addition Levels.

Sample Code	Preparation Conditions (°C, min)	Addition Level
GSP-P ^1^	-	0.6 g/L
GSP-120A ^2^	12,060	0.6 g/L
GSP-150B ^3^	150,180	0.6 g/L

^1^ GSP-P refers to the purified GSP prepared in Section 2.3 without high-pressure reactor treatment. GSP-P is the unautoclaved pure GSP prepared in Section 2.3, representing the GSP sample with the highest degree of polymerization in this study; ^2^ GSP-120A is the GSP sample with a medium degree of polymerization; ^3^ GSP-150B is the GSP sample with a low degree of polymerization.

**Table 2:** Glossary of Astringency Descriptors.

Sensory Attribute	Phase of Perception	Description
Smooth	Enter and Aftertaste	Pleasant texture similar to silk
Velvety	Enter and Aftertaste	Moderately pleasant texture similar to velvet
Rough	Enter and Aftertaste	Rough texture similar to fine sandpaper
Grainy	Enter and Aftertaste	Sensation of particulate matter on the oral surface
Dry	Enter and Aftertaste	Lack of lubrication or a sensation of dehydration in the mouth
Puckery	Aftertaste	Unconscious reaction involving a shrinking or tightening sensation on the oral surface, attempting to re-lubricate the mouth

**Table 3:** Summary of Mean Polymerization Degrees and Recovery Rates of GSP under Different High-Pressure Treatment Conditions.

Sample Code	mDP	Average Recovery Rate
GSP-P	4.63 ± 0.43 a	-
GSP-80A	3.92 ± 0.08 b	92.40 ± 2.76%
GSP-80B	3.69 ± 0.09 bc	92.01 ± 2.52%
GSP-100A	3.44 ± 0.16 cd	91.29 ± 2.09%
GSP-100B	3.30 ± 0.24 de	91.20 ± 3.44%
GSP-120A	3.29 ± 0.06 de	90.56 ± 0.34%
GSP-120B	3.01 ± 0.06 e	88.70 ± 1.85%
GSP-150A	1.70 ± 0.04 f	88.03 ± 1.57%
GSP-150B	1.31 ± 0.02 g	86.98 ± 1.21%

Different letters in the mDP column indicate significant differences (*p* < 0.05). The recovery rate is calculated as the mass of grape seed proanthocyanidins obtained after high-pressure reactor fractionation divided by the mass of the sample introduced. The numbers in the sample codes indicate the temperature (°C) set during autoclave treatment, and the capital letters A and B represent autoclave treatment for 1 h and 3 h, respectively.

**Table 4:** Proanthocyanidins phenolic content (dimer, trimer) in different treatment groups (mg/g).

	GSP-P	GSP-80A	GSP-80B	GSP-100A	GSP-100B	GSP-120A	GSP-120B	GSP-150A	GSP-150B
A2	0.29 ± 0.10 de	0.50 ± 0.05 d	0.24 ± 0.09 de	0.65 ± 0.01 bc	0.31 ± 0.05 de	0.75 ± 0.05 b	0.42 ± 0.10 cde	0.18 ± 0.08 e	0.93 ± 0.26 a
B1	2.87 ± 0.12 d	3.94 ± 0.18 c	2.38 ± 0.27 e	4.27 ± 0.21 ab	4.39 ± 0.34 a	4.02 ± 0.12 bc	4.17 ± 0.14 abc	1.09 ± 0.13 f	0.13 ± 0.03 g
B2	0.61 ± 0.05 a	0.61 ± 0.08 a	0.66 ± 0.15 a	0.65 ± 0.14 a	0.40 ± 0.07 b	0.70 ± 0.16 a	0.36 ± 0.01 b	0.08 ± 0.01 c	0.05 ± 0.01 c
B3	3.53 ± 0.47 d	5.09 ± 0.41 c	9.96 ± 0.13 a	5.79 + 0.2 b	4.90 ± 0.72 c	6.18 ± 1.03 b	5.05 ± 0.33 c	0.51 ± 0.06 e	0.18 ± 0.03 e
B4	1.28 ± 0.13 cd	1.19 ± 0.18 d	1.48 ± 0.11 bc	1.61 ± 0.10 b	1.3 ± 0.19 cd	2.16 ± 0.21 a	1.63 ± 0.17 b	0.37 ± 0.03 e	0.23 ± 0.04 e
Tri	1.87 ± 0.30 a	1.99 ± 0.28 a	1.73 ± 0.36 ab	1.72 ± 0.19 ab	1.91 ± 0.67 a	1.30 ± 0.14 b	1.48 ± 0.25 ab	0.31 ± 0.05 c	0.096 ± 0.05 c

Different letters in the same row indicate significant differences (*p* < 0.05). A2: Procyanidin A2; B1: Procyanidin B1; B2: Procyanidin B2; B3: Procyanidin B3; B4: Procyanidin B4; Tri: Proanthocyanidin Trimer, the same below.

**Table 5:** Contents of flavan-3-ol monomers (mg/L) in wine samples at different aging periods.

Wine Samples	GC	EGC	C	EC	EGCG	ECG
CK-0M	37.24 ± 5.08 a	91.84 ± 52.30 c	111.21 ± 0.60 abcde	64.47 ± 0.16 bdc	16.77 ± 0.26 abcd	LOD
P-0M	38.33 ± 4.36 a	125.04 ± 6.83 abc	133.55 ± 11.47 a	77.65 ± 4.87 a	16.34 ± 1.96 abcde	LOD
PM-0M	32.91 ± 1.85 abc	112.79 ± 6.37 abc	124.67 ± 10.33 abc	74.86 ± 5.20 ab	15.28 ± 1.85 bcde	4.73 ± 1.02 bcd
PL-0M	37.26 ± 2.67 a	111.43 ± 8.81 abc	125.09 ± 13.74 abc	68.85 ± 6.67 abc	19.34 ± 3.16 ab	5.08 ± 1.17 bcd
CK-1M	30.22 ± 2.04 abcde	104.16 ± 5.74 abc	90.85 ± 10.87 ef	54.19 ± 5.27 def	13.39 ± 2.04 cde	0.61 ± 0.01 e
P-1M	33.05 ± 0.83 abc	125.90 ± 1.91 ab	126.07 ± 12.60 ab	73.49 ± 6.27 ab	14.93 ± 1.84 bcde	5.56 ± 0.42 abcd
PM-1M	28.23 ± 3.66 bcde	97.63 ± 23.56 bc	106.76 ± 13.56 abcdef	64.73 ± 7.08 bdc	11.82 ± 2.97 de	3.09 ± 2.17 de
PL-1M	36.10 ± 2.48 ab	101.93 ± 20.50 abc	120.26 ± 1.59 abcd	65.50 ± 0.56 bc	17.23 ± 0.39 abc	2.92 ± 1.81 de
CK-3M	27.58 ± 6.88 cde	99.36 ± 3.81 abc	80.00 ± 5.09 f	46.23 ± 2.90 f	11.40 ± 1.45 e	0.79 ± 0.00 e
P-3M	25.33 ± 4.35 cde	128.35 ± 10.63 ab	115.86 ± 14.96 abcde	65.33 ± 6.72 bc	14.31 ± 2.09 bcde	3.36 ± 4.43 cde
PM-3M	24.45 ± 1.52 de	112.89 ± 1.49 abc	102.86 ± 9.20 bcdef	61.67 ± 4.00 cde	13.38 ± 2.02 cde	3.84 ± 2.65 cde
PL-3M	31.39 ± 7.70 cde	130.42 ± 7.10 ab	91.16 ± 34.75 ef	52.74 ± 13.21 ef	17.02 ± 5.14 abcd	7.24 ± 0.35 ab
CK-6M	23.13 ± 1.04 de	126.61 ± 2.97 ab	84.08 ± 10.66 f	47.64 ± 4.38 f	14.21 ± 1.31 bcde	LOD
P-6M	22.30 ± 0.78 e	129.15 ± 0.64 ab	97.14 ± 15.15 def	53.44 ± 3.92 ef	14.82 ± 1.15 bcde	8.88 ± 0.17 a
PM-6M	26.20 ± 3.33 cde	133.28 ± 2.30 a	98.73 ± 11.56 cdef	54.55 ± 4.97 def	16.25 ± 0.48 abcde	8.67 ± 0.66 a
PL-6M	31.11 ± 8.85 abcd	119.15 ± 24.90 abc	93.85 ± 15.58 def	49.20 ± 5.44 f	21.00 ± 6.47 a	6.59 ± 2.68 abc

Different letters in the same column indicate significant differences (*p* < 0.05). LOD (Limit of Detection) indicates that the required quantity has not been met.

**Table 6:** Tannin structures of wine samples at different aging periods.

Wine Samples	Total Condensed Tannin Content (mg/L)	Mean Degree of Polymerization (mDP)	Procyanidin Percentage (%PC)	Prodelphinidin Percentage (%PD)	Galloylation Percentage (%G)	Terminal Subunits (%)				Extension Subunits (%)			
						tC	tEC	tECG	tEGC	exC	exEC	exECG	exEGC
CK-0M	303.73 ± 26.37 efg	3.34 ± 0.13 a	83.10 ± 0.44 bc	15.42 ± 0.14 bcd	1.47 ± 0.51 g	24.65 ± 1.17 bc	7.57 ± 0.28 gh	1.38 ± 0.42 e	0.61 ± 0.03 c	5.09 ± 0.51 c	57.08 ± 5.94 bc	0.70 ± 0.09 ef	16.90 ± 1.32 ab
P-0M	422.72 ± 26.37 a	3.10 ± 0.11 bc	85.47 ± 0.94 a	13.20 ± 1.06 def	1.33 ± 0.50 g	35.07 ± 3.84 a	8.89± 0.77 g	0.73 ± 0.02 e	5.86 ± 1.19 a	8.73 ± 0.50 a	82.77 ± 5.52 a	0.80 ± 0.02 def	14.95 ± 0.69 abcde
PM-0M	332.94 ± 4.17 cde	2.89 ± 0.24 cd	81.38 ± 1.96 abcd	10.88 ± 0.65 efg	7.74 ± 1.32 f	24.06 ± 0.21 bc	8.02 ± 0.44 gh	8.85 ± 1.85 cd	2.88 ± 0.21 c	8.37 ± 0.43 a	58.19 ± 3.54 b	0.50 ± 0.05 f	12.00 ± 0.68 def
PL-0M	263.00 ± 15.26 gh	2.48 ± 0.11 e	75.10 ± 1.28 ef	16.09 ± 2.07 abcd	8.81 ± 1.50 def	20.64 ± 1.13 cd	6.47 ± 0.55 h	8.38 ± 1.78 d	2.98 ± 0.12 b	4.20 ± 0.19 d	40.12 ± 1.99 efg	0.06 ± 0.00 g	12.31 ± 0.70 def
CK-1M	206.30 ± 20.98 i	3.30 ± 0.09 ab	81.39 ± 0.92 abcd	17.79 ± 0.86 abc	0.83 ± 0.17 g	15.17 ± 0.79 e	6.60 ± 1.25 h	0.72 ± 0.00 e	1.14 ± 0.06 c	3.45 ± 0.31 e	37.64 ± 3.52 fgh	0.06 ± 0.01 g	12.65 ± 1.18 cdef
P-1M	356.05 ± 22.59 bcd	2.05 ± 0.03 gh	81.73 ± 0.83 abc	7.81 ± 0.16 g	10.46 ± 0.68 cdef	18.21 ± 0.89 de	31.17 ± 0.44 a	12.71 ± 1.66 bcd	LOD	5.25 ± 0.33 bc	49.55 ± 3.71 cd	0.68 ± 0.07 ef	9.98 ± 0.78 f
PM-1M	355.73 ± 8.81 bcd	2.03 ± 0.10 gh	77.34 ± 0.85 bcde	9.79 ± 0.87 fg	12.86 ± 0.07 cde	17.90 ± 2.58 de	28.74 ± 0.37 bc	15.63 ± 0.48 bc	LOD	5.22 ± 0.35 bc	45.65 ± 3.04 def	0.59 ± 0.11 ef	12.38 ± 1.38 def
PL-1M	345.48 ± 20.32 bcde	1.76 ± 0.01 i	73.03 ± 1.31 efg	13.26 ± 0.79 def	13.71 ± 1.87 cd	24.96 ± 2.45 b	28.92 ± 0.32 b	15.62 ± 1.08 bc	LOD	3.41 ± 0.17 e	32.15 ± 1.81 ghi	0.99 ± 0.03 cde	16.28 ± 1.93 abc
CK-3M	188.86 ± 9.53 i	2.71 ± 0.10 d	61.58 ± 1.77 h	19.54 ± 0.56 a	18.88 ± 2.31 ab	9.63 ± 0.03 f	2.79 ± 0.22 i	11.74 ± 2.01 bcd	LOD	1.87 ± 0.16 f	25.79 ± 0.49 i	0.63 ± 0.03 ef	12.72 ± 0.15 cdef
P-3M	344.45 ± 17.80 bcde	2.42 ± 0.08 e	75.45 ± 0.65 def	12.88 ± 0.75 def	11.67 ± 0.57 cdef	16.05 ± 0.61 e	21.41 ± 0.48 e	13.12 ± 0.76 bcd	LOD	4.86 ± 0.26 c	50.20 ± 3.06 bcd	1.17 ± 0.13 bcd	15.84 ± 1.78 abcd
PM-3M	360.16 ± 8.52 bcd	1.98 ± 0.06 ghi	71.18 ± 1.54 fg	8.11 ± 0.32 g	20.71 ± 1.86 a	15.79 ± 0.47 e	22.21 ± 0.92 e	24.37 ± 1.80 a	LOD	5.91 ± 0.33 b	44.04 ± 2.71 def	1.15 ± 0.13 bcd	10.03 ± 0.66 f
PL-3M	313.96 ± 9.34 def	1.88 ± 0.03 hi	74.41 ± 0.48 ef	10.72 ± 0.36 efg	14.87 ± 0.76 bc	16.37 ± 0.61 de	27.26 ± 0.75 bcd	15.04 ± 0.34 bcd	LOD	3.42 ± 0.17 e	35.07 ± 1.63 gh	1.33 ± 0.06 abc	11.84 ± 0.77 ef
CK-6M	251.86 ± 12.13 h	2.09 ± 0.06 gh	68.26 ± 0.77 g	18.93 ± 0.87 ab	12.80 ± 1.06 cde	11.25 ± 0.53 f	17.74 ± 0.54 f	10.02 ± 0.30 cd	3.59 ± 0.03 b	2.29 ± 0.16 f	29.58 ± 2.20 hi	1.35 ± 0.17 abc	13.32 ± 1.61 bcdef
P-6M	386.07 ± 14.47 ab	2.39 ± 0.07 ef	75.65 ± 0.81 def	15.75 ± 0.84 bcd	8.61 ± 0.18 ef	16.45 ± 0.48 de	26.70 ± 0.59 cd	10.65 ± 0.36 bcd	4.49 ± 0.32 ab	5.42 ± 0.29 bc	56.71 ± 2.41 bc	1.33 ± 0.24 abc	17.46 ± 2.01 a
PM-6M	372.98 ± 50.41 bc	2.03 ± 0.11 gh	72.80 ± 4.90 efg	13.36 ± 0.78 def	13.84 ± 5.68 cd	16.64 ± 0.86 de	25.73 ± 0.64 d	17.29 ± 9.41 b	5.50 ± 0.14 a	5.88 ± 0.29 b	46.57 ± 2.34 de	1.70 ± 0.32 a	11.92 ± 0.77 ef
PL-6M	286.47 ± 3.65 fgh	2.18 ± 0.17 fg	76.11 ± 8.09 cdef	14.37 ± 5.60 cde	9.52 ± 4.83 def	16.03 ± 1.36 e	18.47 ± 3.07 f	8.30 ± 4.94 d	4.68 ± 1.12 ab	3.41 ± 0.10 e	40.61 ± 8.29 efg	1.47 ± 0.11 ab	10.10 ± 4.61 f

Different letters in the same column indicate significant differences (*p* < 0.05). LOD (Limit of Detection) indicates that the required quantity has not been met.

**Table 7:** Contents of monomeric anthocyanins (mg/L) in wine samples at different aging periods.

	Dp-3-Glu	Cy-3-Glu	Pt-3-Glu	Pn-3-Glu	Mv-3-Glu	Pn-3-Acglu	Mv-3-Acglu	Pn-3-Coglu	Mv-3-Coglu
CK-0M	4.93 ± 0.05 a	0.44 ± 0.00 a	6.93 ± 0.05 a	4.31 ± 0.16 a	102.49 ± 1.37 a	2.31 ± 0.06 a	33.86 ± 0.65 a	5.50 ± 0.28 a	2.39 ± 0.17 ef
P-0M	4.36 ± 0.18 b	0.41 ± 0.02 bc	6.10 ± 0.11 b	2.69 ± 1.16 bc	88.48 ± 5.12 b	2.03 ± 0.08 b	29.30 ± 0.79 b	4.68 ± 0.24 b	2.28 ± 0.13 f
PM-0M	4.28 ± 0.25 b	0.42 ± 0.02 ab	6.02 ± 0.22 b	3.41 ± 1.16 ab	86.44 ± 5.26 bc	1.97 ± 0.11 b	28.23 ± 0.75 c	4.32 ± 0.10 c	2.18 ± 0.16 f
PL-0M	3.88 ± 0.19 cd	0.39 ± 0.02 cd	5.64 ± 0.24 c	2.70 ± 1.10 bc	81.10 ± 4.71 de	1.73 ± 0.29 c	26.48 ± 1.15 d	3.90 ± 0.32 de	2.47 ± 0.33 ef
CK-1M	3.97 ± 0.06 c	0.41 ± 0.02 bc	5.41 ± 0.07 cd	1.98 ± 0.05 cd	82.41 ± 1.24 cd	1.74 ± 0.05 c	26.90 ± 0.30 d	3.96 ± 0.15 d	3.14 ± 0.23 d
P-1M	3.66 ± 0.14 d	0.37 ± 0.02 d	5.15 ± 0.13 e	2.34 ± 0.94 bc	74.73 ± 3.39 f	1.63 ± 0.01 cd	24.81 ± 0.51 e	3.51 ± 0.18 f	3.15 ± 0.23 d
PM-1M	3.91 ± 0.16 c	0.40 ± 0.02 bc	5.45 ± 0.20 cd	2.35 ± 0.90 bc	77.11 ± 4.04 ef	1.54 ± 0.20 d	24.76 ± 0.74 e	3.67 ± 0.08 ef	2.68 ± 0.15 def
PL-1M	3.88 ± 0.20 cd	0.40 ± 0.00 bcd	5.25 ± 0.22 de	2.38 ± 0.90 bc	75.30 ± 2.48 f	1.65 ± 0.06 cd	24.88 ± 0.68 e	3.44 ± 0.16 f	2.94 ± 0.34 de
CK-3M	2.37 ± 0.04 e	0.34 ± 0.02 e	2.86 ± 0.12 f	1.15 ± 0.03 de	54.78 ± 0.52 g	1.10 ± 0.02 d	17.40 ± 0.06 f	2.48 ± 0.13 g	4.12 ± 0.29 c
P-3M	1.77 ± 0.03 g	0.29 ± 0.02 f	2.21 ± 0.05 h	0.84 ± 0.03 de	42.60 ± 0.50 h	0.99 ± 0.04 d	15.03 ± 0.26 g	2.05 ± 0.11 h	4.70 ± 0.44 bc
PM-3M	1.88 ± 0.03 fg	0.30 ± 0.01 f	2.28 ± 0.04 h	0.95 ± 0.03 de	45.20 ± 0.37 h	0.97 ± 0.01 d	15.01 ± 0.08 g	2.10 ± 0.09 h	4.13 ± 0.22 c
PL-3M	2.10 ± 0.26 f	0.30 ± 0.02 f	2.57 ± 0.19 g	1.00 ± 0.06 de	43.59 ± 2.10 h	0.99 ± 0.06 d	14.42 ± 0.72 g	1.87 ± 0.19 h	4.26 ± 0.38 c
CK-6M	0.86 ± 0.02 h	LOD	0.79 ± 0.01 i	0.44 ± 0.01 e	18.83 ± 0.42 i	0.39 ± 0.03 e	5.56 ± 0.15 h	0.89 ± 0.06 i	5.01 ± 0.54 ab
P-6M	0.67 ± 0.02 hi	LOD	0.68 ± 0.02 i	0.39 ± 0.02 e	14.27 ± 0.34 ij	0.39 ± 0.02 e	5.14 ± 0.14 hi	0.78 ± 0.02 i	5.09 ± 0.35 ab
PM-6M	0.60 ± 0.02 i	LOD	0.67 ± 0.01 i	0.38 ± 0.02 e	14.41 ± 0.05 ij	0.35 ± 0.04 e	4.63 ± 0.15 hi	0.78 ± 0.04 i	4.85 ± 0.43 ab
PL-6M	0.61 ± 0.02 i	LOD	0.69 ± 0.03 i	0.40 ± 0.00 e	13.92 ± 0.20 j	0.32 ± 0.02 e	4.54 ± 0.08 i	0.71 ± 0.05 i	5.44 ± 0.52 a

Different letters in the same row indicate significant differences (*p* < 0.05). LOD (Limit of Detection) indicates that the required quantity has not been met.

## Data Availability

The original contributions presented in the study are included in the article. Further inquiries can be directed to the corresponding authors.
